# Expulsion from the Motherland: Association between Depression & Health-Related Quality of Life for Ethnic Rohingya Living with Refugee Status in Bangladesh

**DOI:** 10.2174/1745017902016010046

**Published:** 2020-04-20

**Authors:** K M Amran Hossain, Lori M. Walton, S. M. Yasir Arafat, Nidiorin Maybee, Rubel Hossen Sarker, Shahoriar Ahmed, Feroz Kabir

**Affiliations:** 1 Department of Physiotherapy, Bangladesh Health Professions Institute (BHPI), Savar Union, Bangladesh; 2 Departmnt of Physiotherapy, University of Sharjah, Sharjah, UAE; 3 Department of Psychiatry, Enam Medical College and Hospital, Savar Union, Bangladesh; 4 Department of Physiotherapy, Centre for the Rehabilitation of the Paralysed (CRP), Dhaka, Bangladesh; 5 Department of Rehabilitation, Rohingya Humanitarian Responses, Handicap International (Humanity and Inclusion), Dhaka, Bangladesh; 6 Department of Physiotherapy, Bangladesh Physiotherapy Association (BPA), Dhaka, Bangladesh; 7 Department of Physiotherapy & Rehabilitation, Jashore University of Science & Technology (JUST), Jeshore, Bangladesh

**Keywords:** Rohingya, Refugee, Patient Health Questionnaire (PHQ-9), WHO-QOL BREF, Depression, Psychological state

## Abstract

**Background:**

The Rohingyas are an ethnic minority group from Myanmar who have experienced severe forms of violence such as murder, rape, humanitarian defilement and forcible expellation from their motherland. Exposure to trauma has a long-lasting impact on psychological well-being and Health-related Quality of Life (HRQoL).

**Objective:**

The purpose of this study was to examine the prevalence of depression and association with HRQoL for Rohingya displaced persons.

**Methodology:**

This was a prospective, cross-sectional study in two refugee camps in Southern Bangladesh, with a structured and language validated questionnaire.

**Results:**

The study indicates the prevalence of depression was 70% (n=150 respondents), with 8.7% reporting “severe depression” in PHQ-9. WHOQOL-BREF scores were inversely associated with symptoms on the depression scale with a strong and significant correlation (r= 0.652; p<0.01) in total and physical health; psychological (r= 0.757, p<0.01), social relationship (r= 0.479, p<0.01), environment (r= 0.443, p<0.01), increasing age (r= 0.272, p<0.01), severity of depression (r= 0.489, p<0.01). Furthermore, there was a statistically significant correlation with overall quality of life with same variables subsequently (r =0.600, 0.309, 0.482, 0.170, 0.103, 0.272, 0.339; p<0.01), also correlation was observed between married individuals and severity of depression in PHQ (r= 0.346), physical state (r= 0.353), psychological state (r= 0.358), and with social relationship (r= 0.435), with statistical significance (p= <0.01).

**Conclusion:**

There are higher incidence rates of moderate to severe depression than the population norms and low health-related quality of life than published population norms for Rohingya displaced persons living in refugee camps. Depression rates were inversely associated with HRQoL for Rohingya displaced persons living in refugee camps. Future research may consider the prevention of related medical issues for long term program implementation.

## 1 BACKGROUND

The Rohingyas are an ethnic minority group in the Rakhain state of Myanmar that traces their historical roots in the Arakan region from the eleventh century to 1962 [1, 2]. The United Nations High Commissioner for Refugees (UNHCR) reported, 59.5 million people were forcibly displaced as refugees in 2014 and 65.3 million in 2015 [3-5]. Since 1978, Bangladesh has been working with a humanitarian crisis with the forceful migration of Rohingya from the Rakhine state in Myanmar. Over 1,450,000 refugees have taken shelter in Bangladesh since that time, with the recent influx of over 1,000,000 refugees in 2017 [6-8]. Now, within the community, 50% people are the second generation of Rohingyas, living the majority of their lives in Bangladesh as refugees [9, 10]. Trauma from violence, rape, burning homes, loss of loved ones, forced migration has been experienced by these people. Four hundred and fifty million people suffer from mental health disorders worldwide and among them, 85% are living in low and middle-income countries [11]. The World Health Organization (WHO) reports depression as one of the leading causes for disability worldwide; approximately 7.5% of all years reported under disability in 2015 are because of depression. The report also ranked depression as the major cause of suicide worldwide [11]. The prevalence of depression in Bangladesh constitutes 4.4% of the total population [12]. There is importance examining the physical and mental health status of the refugees for planning programs to meet the needs of men, women and children living with refugee status in Bangladesh. One study of refugees who fled from Myanmar to Thailand border reported the incidence of depression as 41% [13]. UNICEF reports indicate that the Rakhaine state has the second highest rate of poverty, representing 43.5% [8] compared to the World Bank report for Myanmar’s national poverty rate of 32.1% in 2015 [14]. Limited research has been conducted regarding the psychological health of Rohingya Refugees living in Bangladesh. Mental health disorders such as depression, anxiety and stress disorder are projected to be higher among those living with refugee status compared to the general population because of war, trauma, resettlement, migration. Depression and anxiety may persist for a long time after traumatic experiences and may have a direct impact on HRQoL even after the traumatic stimulus is gone [15]. Some studies report depression among refugees, with a higher risk in those with older age, female gender, poor financial status, scattered family, poor living conditions, substandard HRQo Land physical or mental trauma [11, 16]. Studies on refugees showed that unemployment and lack of social relationships are also strong predictors of Low HRQoL [11, 17, 18].There is a need to estimate the overall HRQoL for those living in refugee camps to provide a base for future community driven program assessment. This study seeks to explore the prevalence and association between depression and HRQoL for those living in the refugee camps in Bangladesh.

## 2 METHODOLOGY

To meet up the objectives, a prospective, quantitative, cross-sectional study has been employed at the refugee camp in Cox’s Bazar and Ukhia Health Camp situated in the southern part of Bangladesh from 16^th^ May, 2018 to 17^th^ July, 2018. One hundred fifty (n=150) Rohingya refugees living within two different base camps in the Cox’s Bazar consented to participate in the study. The data was collected through a pretested questionnaire of Health-Related Quality of life WHOQOL-BREF, translated and back translated into Burmese and a Bangla validated Patient Health Questionnaire (PHQ)-9 also translated into Burmese. A Burmese interpreter helped during face to face interviews to gather accurate information from each participant. After obtaining data, oneco-researcher recorded all information in digital form through Microsoft Office 2010 and kept it in a password protected file. All data input was performed by independent data entry personnel to reduce bias. All hard copies were kept secured and confidential and soft copies were kept in a password protected file with the primary investigator. Data were analyzed by an experienced statistician, skillful in analyzing data for prevalence and correlation analysis. Statistical tests for finding correlation was performed by Pearson’s Correlation Coefficient between PHQ scores and WHOQOL scores. Prevalence was calculated for both depression and HRQoL.

## 3 RESULTS

**Fig. (1):**
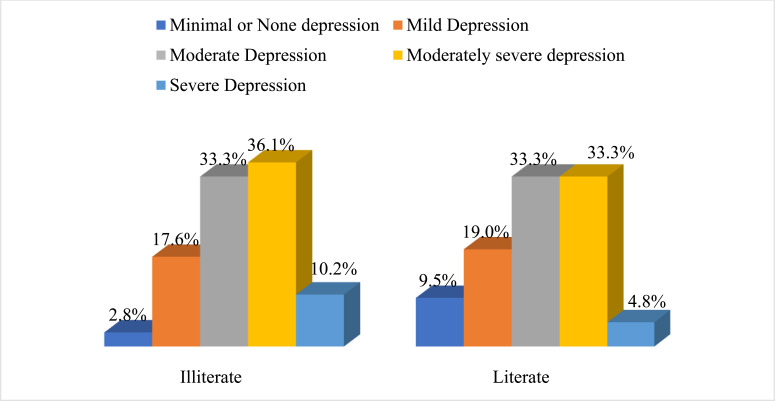
PHQ-9: Patient Health Questionnaire Severity Distribution. Literate has been defined as participants who have at least primary education and can sign his/her name and Illiterate defined by participants who have below primary education and unable to sign his/her name.

Among 150 respondents, 53.3% (n=80) were male and 46.7% (n=70) were female. Most of the participants were in their 3rd decade of life that consisted of 22.7% (n=34), followed by 20% (n=30) in between 40-49 years and 15.3% (n=23) in between 50-59 years. Sixty-five percent (n=98) of the respondents were married and 20% (n=30) were unmarried and 14.7% (n=22) reported widow status. Among all respondents, 72% (n=108) participants reported literacy challenges and 28% (n=42) reported literacy. Forty percent (n=61) participants identified as housewives and 28% (n=42) participants reported “unemployed status”; 51.3% (n=77) lived with their nuclear family and 48.7% (n=73) lived with extended family (Table **1**). The majority of the participants (35.3%) had moderately severe depression; 33.3% had moderate depression; 18% had mild depression; 4.7% had minimal depression; and 8.7% had severe depression according to the PHQ-9 and health questionnaire. The severity was calculated based on the PHQ-9 scale score distribution as mentioned; 0-4 minimal depression, 5-9 mild depression, 10-14 moderate depression, 15-19 moderately severe depression, and 20-27 severe depression [19, 20]. Relatively, 108 respondents reported having literacy challenges; among those who identified as having literacy challenges, 26% reported moderately severe depression and 7.3% reported severe depression. Forty-two respondents identified as literate, among them,the majority of the participants also reported moderate depression and moderately severe depression, with21% and 3%, respectively (Fig. **1**). Moreover, 96 respondents were married; among them, the majority of the participants had moderate depression (54%) and severe depression was 3%. Thirty respondents were unmarried; among those who identified as unmarried, 21% reported having moderately severe depression and 4.5% reported severe depression. Twenty-four respondents were widowed, and 12% reported moderately severe depression and 12% reported severe depression. Minimal depression was only reported in married women.

In this study, theme an overall HRQoL found 1.75±0.87, revealed that 95% of respondents lead “very poor” to “neither poor nor good” quality of life state (p<0.001). Similarly, most of the refugees are dissatisfied with health 2.13±0.85 (P= <0.001). Mobility (1.90 ± 0.86) was also rated low by the sample, reflecting a need for most of the refugees to have more room for “mobility”. Mentioning the psychological state, poorer life enjoyment, meaningless of life, self-esteem and negative feeling have a very strong association (p<0.001). The social relationship and environment were neither “good” nor “satisfactory level”, indicating social and environmental challenges.

There is strong correlation (p<0.01) among WHO QOL total and physical health (r= 0.652), psychological (r= 0.757), social relationship (r= 0.479), environment (r= 0.443), increasing age (r= 0.272), severity of depression (r= 0.489). Moreover, there are significant correlation with overall quality of life with physical health (r= 0.309), psychological state (r= 0.482), social relationship (r= 0.170), increasing age (r= 0.272) and depression (r= 0.339) with statistical significance (P= <0.05) (Table **2**). Furthermore, there is a correlation between married individuals with severity of depression in PHQ (r= 0.346, p= <0.01), depression in PHQ with physical state (r= 0.353, p= 0.<01), psychological state (r= 0.358, p= <0.01), and social relationship (r=435, p= <0.01) (Table **3**).

## 4 DISCUSSION

The study was intended to find out the prevalence, level of depression and associated quality of life in Rohingya Refugees living in Bangladesh. As the respondents were 9 to 86 years old, the demographics revealed diverse facts upon their socio-cultural diversities. The study revealed, most of the migrant refugees in Bangladesh border were in active and productive age period (30-49) and male or female migrants were more or less the same [11]. The majority of the refugees reported literacy challenges. This was not surprising, as Rohingyas receive almost no support for basic education in Myanmar, nor in Bangladesh [21, 22]. The occupations of most of the females identified as housewives and males worked in agriculture or labor-based occupations. This is similar to a study that [22] found Rohingya refugees on the Thai border reported agriculture or labor-based occupations. In the refugee camps, more than half of the migrants lived without family, we speculate this may be because of lost family members in the violence prior to migrating from Rakhaine [8, 22-24]. UNHCR states, now 50% of the total Rohingyas are living in Rakhaine and another 50% migrated to neighboring countries.

Exploring the depression level, almost 70% of Rohingya refugees reported moderately severe depression and 8.7% lived with severe depression state with devastating thoughts about life. The cumulative percentage of moderate to severe depression is 77.3% among Rohingya Refugees. This situation has clinical indications for the management of depression. Depression and age were found to be strongly associated, with the active young adult facing the worst form of depression. The state for those who identified as illiterate, female or a widow was even more dismal. The study exposes the emerging demand for psychological support, aid and treatment for mental health issues for more than 8 out of 10 Rohingya refugees living in Bangladesh [10, 25, 26]. The psychological state and depression level are the worst situations among all the refugees in the world in comparison to Afghanistan, Syria, West Africa and Iraq [27, 28]. To estimate the depression level with several psycho-social components, this study found inverse relationships betweenliteracy level and marriage status for women, suggesting lower rates of marriage for more educated women [12]. This study also suggested similar findings inverse relationship between marriage status and depression. The widow was found to be more severely depressed, with “minimum depression” found more in married people and “moderately severe” depression found in mostly unmarried refugees, suggesting the social/family component to be a strong facilitator of resilience in the midst of traumatic experiences. Females reported more “severe depression” state than males, we speculated this may be because of the higher report of sexual violence, rape and ethnic cleansing acts against females compared to males in the study [10, 28, 29].

The Patient Health Questionnaire (PHQ-9) was used to determine the level of depression among Rohingyar efugees. A similar study on refugees was conducted in Germany to determine the Psychological state for Arabic speaking refugees [27]. WHO-QOL Brief Questionnaire was used to determine the HRQoL associated with depression, similar to a study that used WHO-QOL Brief revealed HRQoL among migrated Iranian refugees [30].

The Rohingya refugees living in Bangladesh reported living the worst estimated health-related quality of life category determined by the World Health Organization. The majority of Rohingyas reported leading poor quality of life, ranging from “very poor” to “neither good nor poor” state, with none of the respondents reporting a normal HRQoL. There were no studies available on HRQoL for Rohingya living in Bangladesh, with very little reported about their state in Rakhaine. UNHCR and the World Bank reported Rakhaine to have the highest open defecation rate, lowest primary education and parental care constituting 40.7%, 31.7% and 1.3% [10, 14]. The study reports indicate that Rohingyas were mostly dissatisfied with their health-related state. The refugees in Bangladesh reported a higher level of depression and lower health-related quality of life compared to norm standard scores across all age groups. Future research should consider mental health support programs focused on improving literacy for women who live within the refugee camps, focusing on social programs and environmental changes to facilitate resilience and reduce depression, specifically in unmarried women, with low literacy abilities.

## CONCLUSION

This study explored a higher than the standard prevalence of depression and a strong negative correlation with health-related quality of life among a group of ethnic Rohingya Muslims living as refugees in Southern Bangladesh. This study indicates a need for mental and social health services to be focused on providing mental health counseling, social programs and environmental change assessment for refugees living in the Rohingya camps in Bangladesh.

## Figures and Tables

**Table 1:** Distribution of demographic variables of the respondents (n=150).

**Demographic Variable**	**Frequency (n)**	**Percentage (%)**
**Age range**		
<10 y	1	0.7
10-19 y	17	11.3
20-29 y	20	13.3
30-39 y	34	22.7
40-49 y	30	20.0
50-59 y	23	15.3
60-69 y	15	10.0
70-79 y	8	5.3
>80 y	2	1.3
**Gender**		
Male	80	53.3
Female	70	46.7
**Marital Status**		
Married	96	64.0
Unmarried	30	20.0
Widow	24	16.0
**Education**		
Illiterate	108	72.0
Literate	42	28.0
**Occupation**		
Fisher Man	1	0.7
Agriculture	3	2.0
Driver	2	1.3
Day laborer	12	8.0
Unemployed	42	28.0
Housewife	61	40.7
Student	13	8.7
Other	16	10.7
**Family Type**		
Lives without family	77	51.3
Lives with family members	73	48.7

**Table 2:** Correlation between WHOQOL-BREF total and domain scores with other measures (n =150).

		WHOQOL Total	QOL Overall	Physical Health	Psychological	Social Relationship	Environment	Age Range	Severity of Depression
WHOQOL Total	r-value	1	0.600**	0.652**	0.757**	0.479**	0.443**	-0.272**	-0.489**
	P-value		0.000	0.000	0.000	0.000	0.000	0.001	0.000
QOL Overall	r-value	0.600**	1	0.309**	0.482**	0.170*	0.103	-0.272**	-0.339**
	P-value	0.000		0.000	0.000	0.038	0.210	0.001	0.000
Physical health	r-value	0.652**	0.309**	1	0.323**	0.271**	-0.016	-0.214**	-0.285**
	P-value	0.000	0.000		0.000	0.001	0.842	0.008	0.000
Psychological	r-value	0.757**	0.482**	0.323**	1	0.324**	0.042	-0.219**	-0.526**
	P-value	0.000	0.000	0.000		0.000	0.611	0.007	0.000
Social Relationship	r-value	0.479**	0.170*	0.271**	0.324**	1	-0.042	-0.045	-0.439**
	P-value	0.000	0.038	0.001	0.000		0.607	0.588	0.000
Environment	r-value	0.443**	0.103	-0.016	0.042	-0.042	1	-0.068	0.042
	P-value	0.000	0.210	0.842	0.611	0.607		0.411	0.607
Age range	r-value	-0.272**	-0.272**	-0.214**	-0.219**	-0.045	-0.068	1	0.040
	P-value	0.001	0.001	0.008	0.007	0.588	0.411		0.625
Severity of Depression	r-value	-0.489**	-0.339**	-0.285**	-0.526**	-0.439**	0.042	0.040	1
	P-value	0.000	0.000	0.000	0.000	0.000	0.607	0.625	
**. Correlation is significant at the 0.01 level (2-tailed).									
*. Correlation is significant at the 0.05 level (2-tailed).									

**Table 3:** Correlation among variables.

		Occupation	Family Type	Gender	Age Range	PHQ Severity	Physical	Psychological	Social Relationship	Environment
Marital Status	Pearson Correlation	0.178*	-0.035	0.170*	-0.061	0.346**	-0.009	-0.123	-0.105	-0.036
	Sig. (2-tailed)	0.029	0.674	0.038	0.456	0.000	0.912	0.134	0.201	0.665
	N	150	150	150	150	150	150	150	150	150
Education	Pearson Correlation	0.205*	-0.102	-0.018	0.062	-0.128	0.084	-0.052	0.167*	-0.112
	Sig. (2-tailed)	0.012	0.213	0.828	0.449	0.118	0.309	0.530	0.042	0.173
	N	150	150	150	150	150	150	150	150	150
Gender	Pearson Correlation				0.226**	0.057	-0.077	0.021	-0.024	0.038
	Sig. (2-tailed)				0.005	0.489	0.347	0.802	0.774	0.642
	N				150	150	150	150	150	150
Age_Range	Pearson Correlation					0.058	-0.097	-0.186*	-0.034	-0.033
	Sig. (2-tailed)					0.483	0.238	0.023	0.682	0.691
	N					150	150	150	150	150
PHQ_Severity	Pearson Correlation						-0.353**	-0.358**	-0.435**	0.150
	Sig. (2-tailed)						0.000	0.000	0.000	0.066
	N						150	150	150	150
Physical	Pearson Correlation							0.307**	0.512**	-0.006
	Sig. (2-tailed)							0.000	0.000	0.947
	N							150	150	150
Psychological	Pearson Correlation								0.317**	0.186*
	Sig. (2-tailed)								0.000	0.022
	N								150	150

*. Correlation is significant at the 0.05 level (2-tailed) **. Correlation is significant at the 0.01 level (2-tailed).
